# Nonnegative matrix factorization analysis and multiple machine learning methods identified IL17C and ACOXL as novel diagnostic biomarkers for atherosclerosis

**DOI:** 10.1186/s12859-023-05244-w

**Published:** 2023-05-12

**Authors:** Li Rao, Bo Peng, Tao Li

**Affiliations:** 1 Department of Geriatrics, Renmin Hospital of Wuhan University, Wuhan, 430060 Hubei China; 2 Department of Cardiology, Renmin Hospital of Wuhan University, Wuhan, 430060 Hubei China; 3 Cardiovascular Research Institute of Wuhan University, Wuhan, 430060 Hubei China; 4 Hubei Key Laboratory of Cardiology, Wuhan, 430060 Hubei China; 5 Department of Neurology, Renmin Hospital of Wuhan University, Wuhan, 430060 Hubei China

**Keywords:** Atherosclerosis, IL17C, ACOXL, Machine learning, Immune infiltration

## Abstract

**Background:**

Atherosclerosis is the common pathological basis for many cardiovascular and cerebrovascular diseases. The purpose of this study is to identify the diagnostic biomarkers related to atherosclerosis through machine learning algorithm.

**Methods:**

Clinicopathological parameters and transcriptomics data were obtained from 4 datasets (GSE21545, GSE20129, GSE43292, GSE100927). A nonnegative matrix factorization algorithm was used to classify arteriosclerosis patients in GSE21545 dataset. Then, we identified prognosis-related differentially expressed genes (DEGs) between the subtypes. Multiple machine learning methods to detect pivotal markers. Discrimination, calibration and clinical usefulness of the predicting model were assessed using area under curve, calibration plot and decision curve analysis respectively. The expression level of the feature genes was validated in GSE20129, GSE43292, GSE100927.

**Results:**

2 molecular subtypes of atherosclerosis was identified, and 223 prognosis-related DEGs between the 2 subtypes were identified. These genes are not only related to epithelial cell proliferation, mitochondrial dysfunction, but also to immune related pathways. Least absolute shrinkage and selection operator, random forest, support vector machine- recursive feature elimination show that IL17C and ACOXL were identified as diagnostic markers of atherosclerosis. The prediction model displayed good discrimination and good calibration. Decision curve analysis showed that this model was clinically useful. Moreover, IL17C and ACOXL were verified in other 3 GEO datasets, and also have good predictive performance.

**Conclusion:**

IL17C and ACOXL were diagnostic genes of atherosclerosis and associated with higher incidence of ischemic events.

**Supplementary Information:**

The online version contains supplementary material available at 10.1186/s12859-023-05244-w.

## Background

Atherosclerosis (AS) is a chronic progressive inflammatory disease of blood vessels, which involves physiological processes such as disorders of lipid metabolism, vascular endothelial cell injury, inflammatory cell infiltration, and neo-capillary formation [1–3]. AS has no obvious symptoms in the early stage of the disease, and patients are mostly aware of it because of other cardiovascular and cerebrovascular complications [4]. Although drugs are available to treat atherosclerosis, there are still many patients who do not benefit from current drug therapy without significant effects or who are intolerant to adverse effects [5]. The search for highly sensitive and specific biomarkers can help reduce the morbidity and mortality of AS. However, current timely diagnostic biomarkers for AS are very limited and not well suited for the early and accurate diagnosis of AS. Therefore, it is important to find new diagnostic markers of AS for accurate diagnosis of AS.

Currently, the development of microarrays has led to extensive and in-depth analysis of genome-wide mRNA expression profiles. With the rapid development of gene chips, high-throughput sequencing, multi-omics analysis and other technologies, gene expression public databases are rapidly increasing. With the development and maturation of bioinformatics, bioinformatics techniques are widely used to analyze large number of expression profiling microarrays to find biomarkers related to disease diagnosis, treatment and prognosis [6–8]. Machine learning has been widely used in finding markers for disease diagnosis base on multi-omics analysis. Support vector machine (SVM), least absolute shrinkage and selection operator (LASSO) regression and random forest (RF) methods are 3 important techniques in machine learning [9–11]. Due to the three methods can identify the best classification feature factor and build a prediction model with generalizability and high prediction accuracy. Xiong et al. screened 2 mRNAs as potential diagnostic biomarkers for abdominal aortic aneurysm using machine learning [12].

In this study, we used multiple machine learning methods analysis to analyze the expression profile microarrays of AS, aiming to screen genes closely related to AS diagnosis and provide new genetic diagnostic markers for AS. We firstly identified 2 subtypes of atherosclerosis by using nonnegative matrix factorization (NMF) algorithm in GSE21545 dataset, and identified prognosis-related DEGs between the subtypes. After that, we identified 2 potential diagnosis genes by using LASSO, RF, SVM-RFE methods and developed a novel prediction model for AS diagnosis. We validated the model and found that the novel prediction model achieved a high AUC in 3 validation AS cohort.

## Materials and methods

### Data collection and pre-processing

The gene expression profiles of human atherosclerosis samples and healthy control samples were obtained from array-based data available in the Gene Expression Omnibus (GEO) database. The search strategy aimed to find published dataset which included a three-step search strategy that was carried out from inception to July, 2022. An initial limited search using the keywords: “Atherosclerosis”, “Atheromatosis”, “Homo sapiens”, “Expression profiling by array”. Dataset needs to meet the following points: (1) Homo sapiens; (2) Atherosclerosis; (3) Sample size greater than 20 cases. Four microarray data sets (GSE21545 [13], GSE20129 [14], GSE43292 [15], GSE100927 [16]) were utilized in our analysis (Additional file 1: Fig. S1). From the GSE21545 dataset, 126 AS samples were included. From GSE20129, 71 control samples and 48 AS samples were included. From GSE43292, the 32 control samples and 32 AS samples were included. From GSE100927, 35 control samples and 69 AS samples were included. The training set was obtained from GSE21545, and the validation set was obtained from GSE20129, GSE43292, GSE100927. The raw files from the four datasets were pre-processed and normalized using limma or RMA-affylmGUI in R Bioconductor.

### Nonnegative matrix factorization (NMF) analysis in GSE21545

The R package “NMF” was performed to identify molecular subtypes based on the gene expression profiles, and patients were classified for follow up studies. We used a NMF algorithm to determine the number of clusters and their stability according to parameters such as cophenetic, dispersion, silhouette, and sparseness [17].

### Identification of differentially expressed genes (DEGs) and prognosis genes between subtypes

DEGs between subtypes were identified using the R package limma with screening criteria of adjusted *P* value < 0.05 [18]. Then, we performed univariate COX analysis to determine the prognostic value of each DEGs.

### Functional and pathway enrichment analysis

To explore the biological functions mainly performed by DEGs and prognosis genes between subtypes, we performed functional enrichment analysis using the "clusterProfiler" package, including GO and KEGG analysis. The screening criteria were *P* < 0.05 and FDR < 0.05 [19, 20].

### Evaluation of immune infiltrating cells in AS

Based on the normalized gene expression data from the disease and control samples, the web tool CIBERSORT (http://CIBERSORT.stanford.edu/) was used to calculate immune cell infiltration and explore the disease immune microenvironment. The 22 immune cell genes (LM22) were used as the reference set. The number of permutations set was 1000. A *P* value < 0.05 in the CIBERSORT results was retained [21].

### Machine learning methods

LASSO method, which is suitable for the reduction in high-dimensional data, was used to select the optimal predictive features in risk factors from the patients with AS. Support vector machine-recursive feature elimination (SVM-RFE) approach is based on the VC dimensional theory of statistical learning theory and the structural risk minimization principle. Based on limited sample information, SVM-RFE seeks to find the best compromise between the complexity of the model (the learning accuracy) and the learning ability. Random forest (RF) refers to a classifier that uses multiple trees to train and predict samples. The three classifiable models’ overlapping genes were then figured out.

### Construction of AS diagnosis nomogram

We used the expression level of predictors by the R-package “rms” to construct the nomogram and predict the risk of AS. Calibration curves were used to estimate the consistency between predicted and actual diagnosis, and the performance of the model in predicting diagnosis was evaluated by area under curve (AUC) [22].

### Statistical methods

R software (4.1.2) was employed to carry out all statistical analysis and graph plotting. Wilcoxon test was applied to analyze the differences between two groups. Kruskal–Wallis test was used for comparison among more than two groups of samples. The Kaplan–Meier method was used to plot survival curves for prognostic analysis, and the log-rank test was used to determine the significance of differences. The correlation test was performed using Spearman correlation analysis and distance correlation analysis. Comparisons of composition ratios among groups were performed by chi-square test. All statistical P values were two-tailed, and *P* < 0.05 was used as the truncated value.

## Results

### Identification of molecular subtypes in AS

**Fig. 1:**
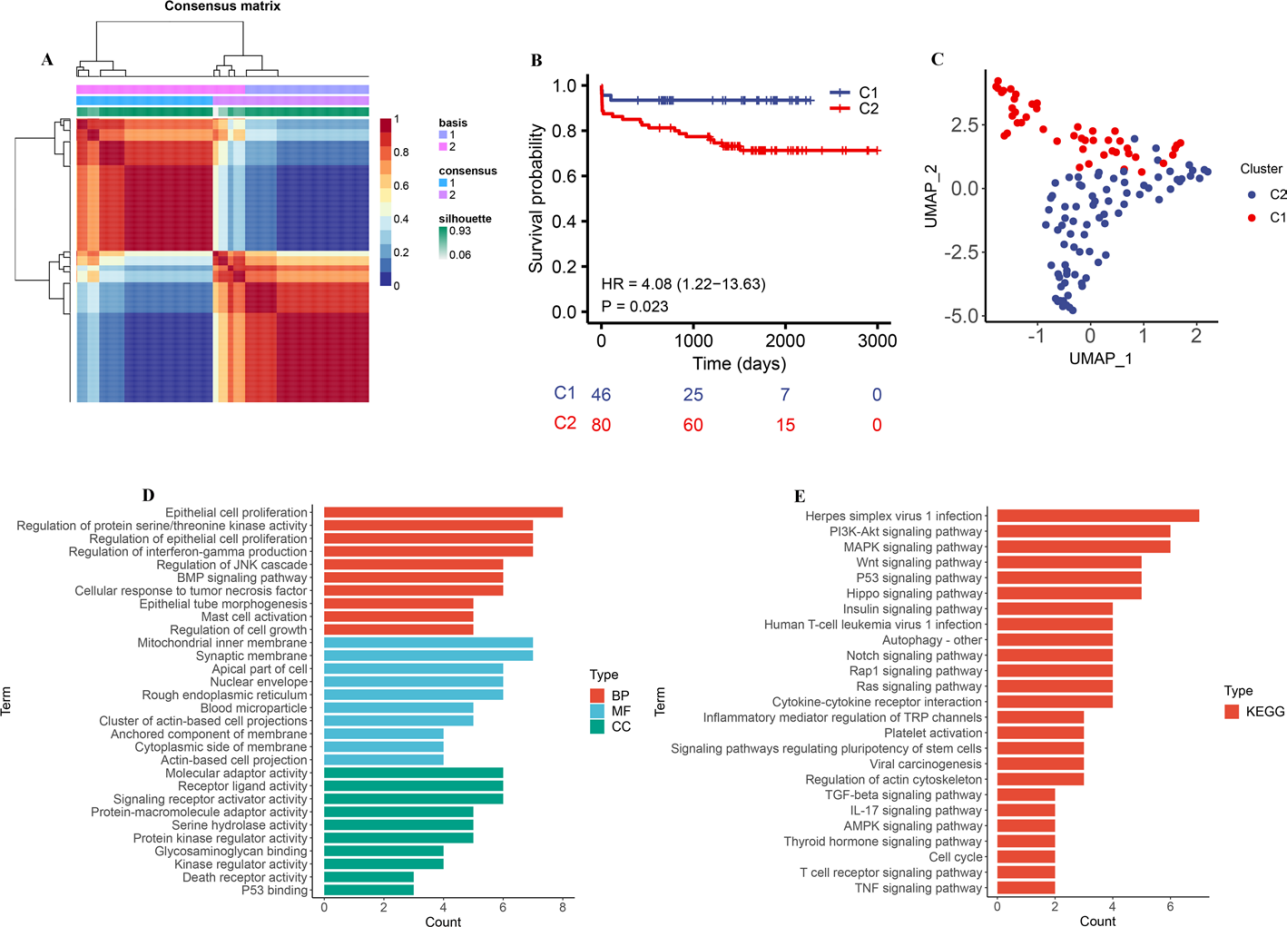
The molecular subtypes categorization of AS base on expression profiles. (**A**) AS patients from GSE21545 dataset were divided into sub‐consensuses based on the gene transcriptional profiling using NMF method. Consensus maps showed the correlation profiling of AS derived from two sub‐consensuses. (**B**) Kaplan–Meier curve showed a significant difference between the two clusters. (**C**) UMAP analysis for the transcriptome profiles of C1 cluster and C2 cluster, showing a remarkable difference on transcriptome between different group. (**D**) GO enrichment analysis, (E) KEGG enrichment analysis for prognostic related DEGs

To explore the expression characteristics in AS, we qualitatively classified patients based on the expression profiles. By NMF algorithm, a cluster number of 2 was the best choice to classify the whole sample into C1 (n = 46) and C2 (n = 80) in GSE21545 dataset (Additional file 2: Fig. S2, Fig. 1A). Kaplan–Meier survival analysis indicated that patients with RPMRs.cluster.A had a worse ischemic events ((HR 4.08, 95% CI 1.22–13.63, *P* = 0.023, Fig. 1B). To explore the potential biological change between distinct cluster, firstly, the PCA demonstrated there is significant DEGs between the two clusters, and 223 prognostic related DEGs were identified (Fig. 1C, Additional file 3: Table S1). Then, we applied GO and KEGG enrichment analysis, which showed that C2 was significantly enriched in immune-related pathways (mast cell activation, regulation of interferon-gamma production, and IL-17 signaling pathway), epithelial cell proliferation (epithelial cell proliferation, regulation of epithelial cell proliferation), mitochondrial dysfunction (mitochondrial inner membrane), suggesting that C2 may play an important role in AS development and immune regulation (Fig. 1D, E).

### Immune infiltrating cell analysis between the two molecular subtypes

**Fig. 2:**
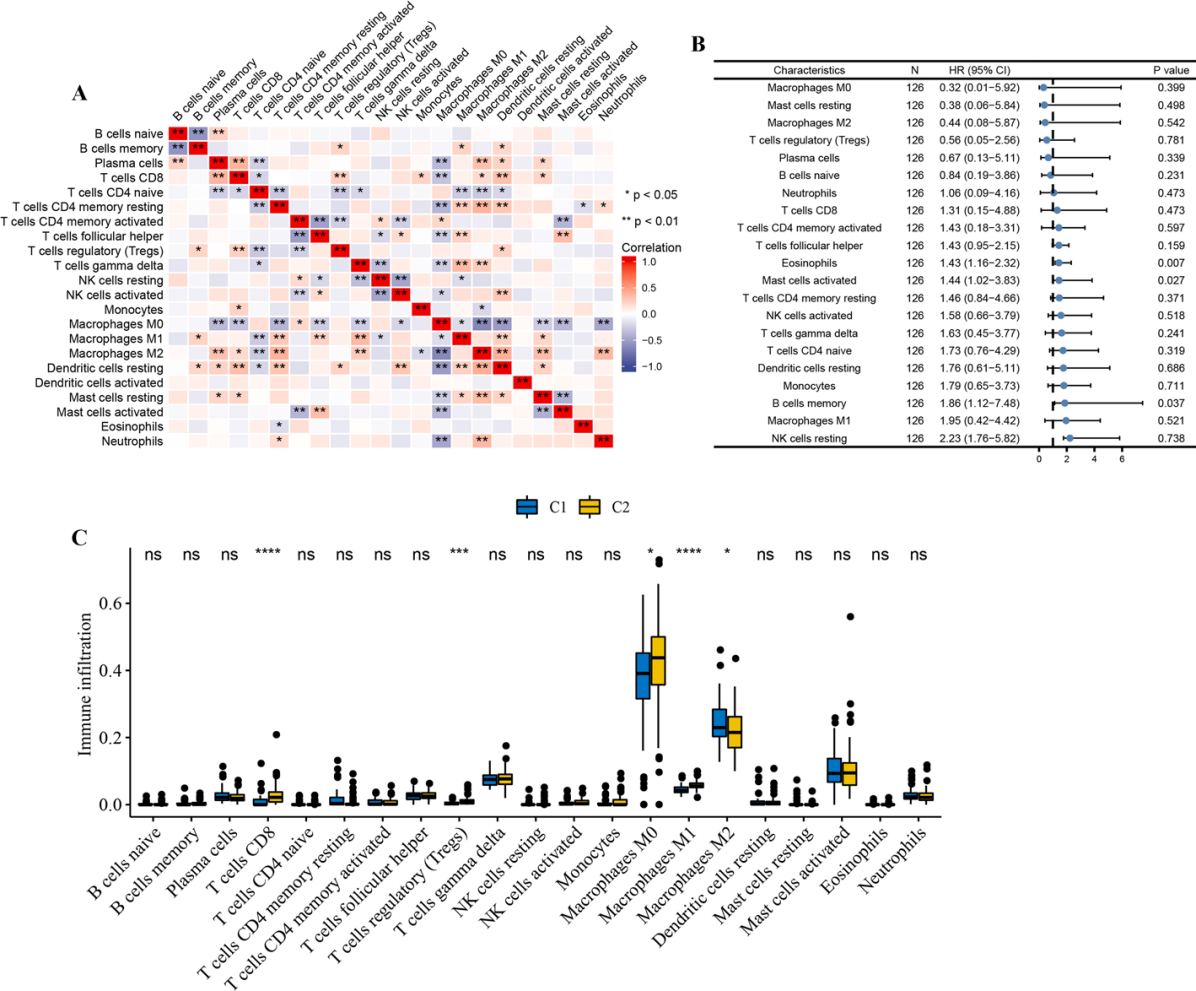
Immune infiltrating cell analysis between the two molecular subtypes. (**A**) Correlation heat map of immune infiltrating cell. The size of the colored squares represents the strength of the correlation; blue represents a negative correlation, and red represents a positive correlation. The darker the color is, the stronger correlation is. (**B**) Forest plots showing the results of the univariate Cox regression between immune infiltrating cell and ischemic events in AS. (**C**) The abundance of each immune infiltrating cell in two clusters. The upper and lower ends of the boxes represented the interquartile range of values. The lines in the boxes represented median value, and black dots showed outliers. (*P < 0.05; ** P < 0.01; *** P < 0.001; **** P < 0.0001)

To further investigate the role of two cluster in immune infiltrating of AS, we used CIBERSORT to explore the infiltration of various types of immune cells in AS samples. Figure 2A indicated the immune cell infiltration landscape and immune cell score correlation results in different samples of the GSE21545 dataset, respectively. Moreover, univariate Cox regression analysis was performed based on GSE21545 dataset, high immune cell score of eosinophils, mast cells activated, B cells memory corelated with worse ischemic events in AS (Fig. 2B). In addition, we also evaluate the association between molecular subtypes and immune cell subpopulations. The results showed T cells CD8, T cells regulatory (Tregs), M0 macrophages, and M1 macrophages were more abundant in C2 cluster, while M2 macrophages was significantly higher in C1 cluster (Fig. 2C). The above results suggest that C2 cluster had a higher inflammatory environment, which leads to the progression of the disease.

### Machine learning methods to detect diagnostic markers in AS

**Fig. 3:**
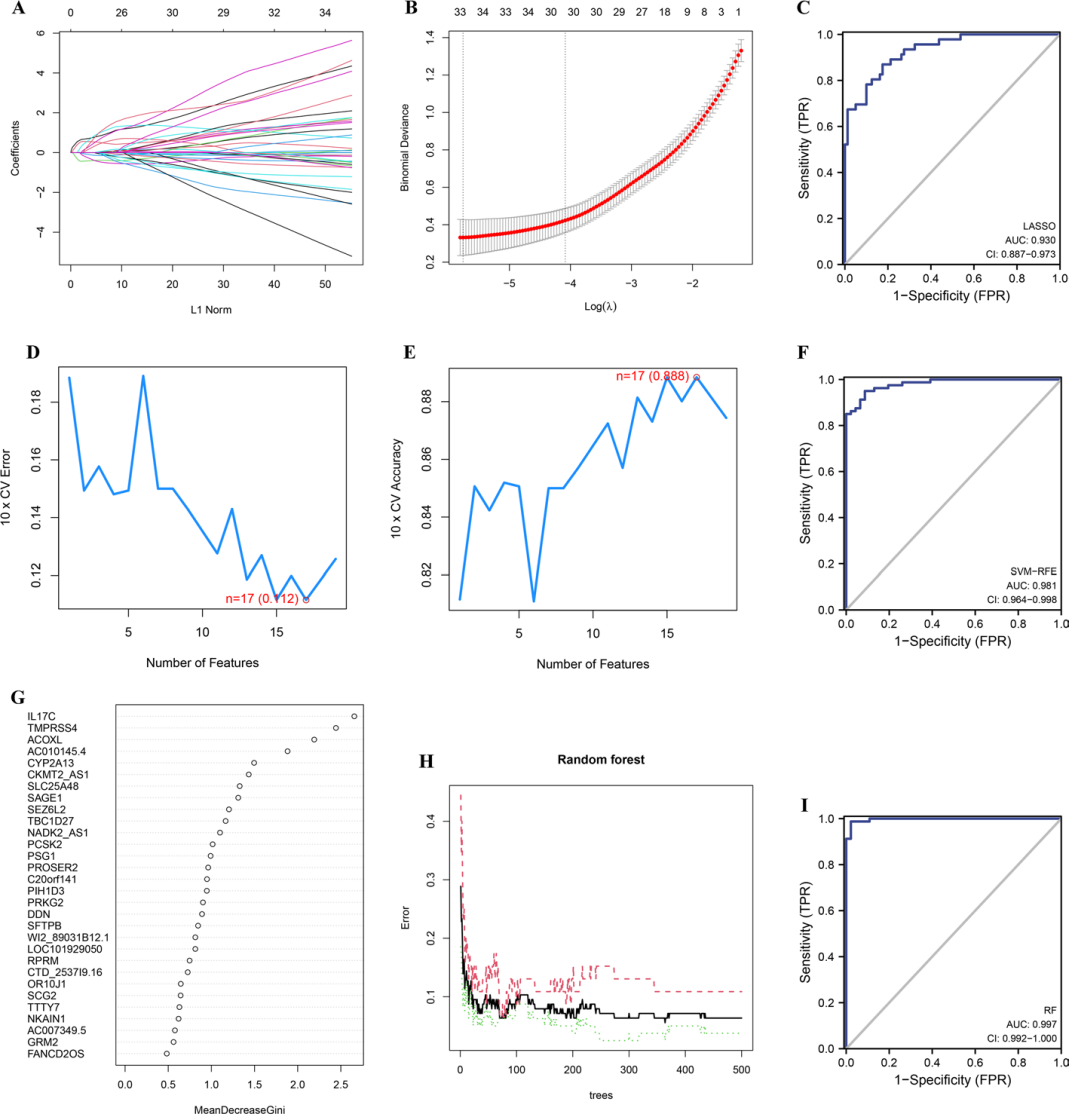
Machine learning methods to detect diagnostic markers in AS. (**A**) Fine-tuning the least absolute shrinkage and selection operator (LASSO) model’s feature selection. (**B**) LASSO regression was used to narrow down the prognostic related DEGs, resulting in the discovery of 33 variables as potential markers for AS. The ordinate represents the value of the coefficient, the lower abscissa represents log (λ), and the upper abscissa represents the current number of non-zero coefficients in the model. (**C**) ROC curves were constructed to assess the diagnostic accuracy of the LASSO model. (**D**, ** E**) A plot illustrating the process of selecting biomarkers using the SVM-RFE technique. The SVM-RFE technique was used to identify a subset of 17 characteristics from the prognostic related DEGs. (**F**) ROC curves were constructed to assess the diagnostic accuracy of the SVM-RFE model. (**G**) The Gini coefficient method’s results in a random forest classifier. The x-axis displays the genetic variable, and the y-axis the significance index. (**H**) The effect of the decision tree number on the error rate. The x-axis denotes the number of decision trees, while the y-axis shows the error rate. (**I**) ROC curves were constructed to assess the diagnostic accuracy of the RF model

To further explore the risk gene features, we carried out the LASSO regression model to screen out 33 potential predictors from 223 prognostic related DEGs (Fig. 3A, B, Additional file 4: Table S2). To evaluate the discrimination of the prediction model, the AUC of ROC was estimated. As shown in Fig. 3C, the prediction model achieved a AUC of 0.930 (95% CI 0.887–0.973), which indicated good discrimination of the model. Then, we carried out the SVM-RFE approach (k = 10, halve.above = 50) to screen out 17 potential predictors from 223 prognostic related DEGs (Fig. 3D, E, Additional file 5: Table S3). To evaluate the discrimination of the prediction model, the AUC of ROC was estimated. As shown in Fig. 3F, the prediction model achieved a AUC of 0.981 (95% CI 0.964–0.998), which indicated good discrimination of the model. In addition, we carried out the RF approach (ntree = 500) to screen out 3 potential predictors from 223 prognostic related DEGs (Fig. 3G, H, Additional file 6: Table S4). To evaluate the discrimination of the prediction model, the AUC of ROC was estimated. As shown in Fig. 3I, the prediction model achieved a AUC of 0.997 (95% CI 0.992–1.000), which indicated good discrimination of the model. The above results show that the three machine learning methods have good performance in identifying diagnostic markers.

### IL17C and ACOXL were identified as diagnostic biomarkers for AS

**Fig. 4:**
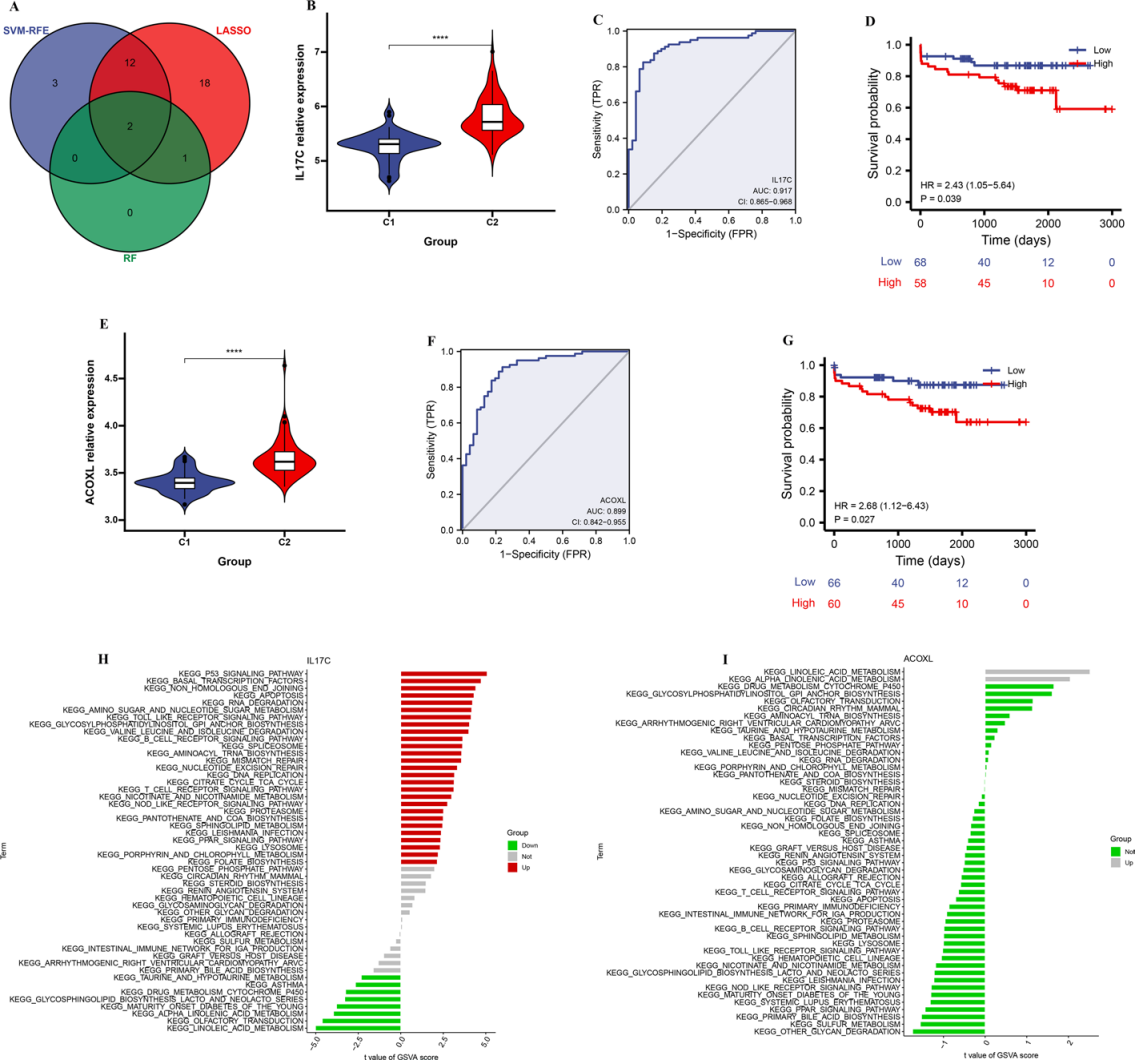
Diagnostic indicators for AS screening. (**A**) Venn diagram showing overlapping markers. (**B**) The illustration shows the expression distribution of IL17C between C2 cluster (red) and C1 cluster (blue). (**C**) ROC curves were constructed to assess the diagnostic accuracy of the IL17C. (**D**) Kaplan–Meier curve showed a significant difference between the high and low IL17C exprseeion. (**E**) The illustration shows the expression distribution of ACOXL between C2 cluster (red) and C1 cluster (blue). (**F**) ROC curves were constructed to assess the diagnostic accuracy of the ACOXL. (**G**) Kaplan–Meier curve showed a significant difference between the high and low ACOXL exprseeion. (**H**) KEGG enrichment analysis for IL17C. (**I**) KEGG enrichment analysis for ACOXL

To further explore the diagnostic biomarkers for AS, the three classifiable models’ overlapping genes were figured out (Fig. 4A). These genes included IL17C and ACOXL. Next, we compared the expression of IL17C and ACOXL in two molecular subtypes, of which the expression level of IL17C and ACOXL were significantly higher in C2 cluster than in C1 cluster (Fig. 4B, E). To evaluate the discrimination of the diagnostic biomarkers, the AUC of ROC was estimated. The IL17C achieved a AUC of 0.917 (95% CI 0.865–0.968) (Fig. 4C) and the ACOXL achieved a AUC of 0.899 (95% CI 0.842–0.955) (Fig. 4F), which indicated good discrimination of the diagnostic biomarkers. Next, Kaplan–Meier survival analysis was performed based on GSE21545 dataset, high expression of IL17C coorelated with worse ischemic events ((HR 2.43, 95% CI 1.05–5.64, *P* = 0.039, Fig. 4D), high expression of ACOXL coorelated with worse ischemic events (HR 2.68, 95% CI 1.12–6.43, *P* = 0.027, Fig. 4G). Then, we applied KEGG enrichment analysis, which showed that high expression IL17C was significantly enriched in immune-related pathways (B cell receptor signaling pathway, T cell receptor signaling pathway), metabolic pathways (citrate cycle TCA cycle, gylcosylphosphatidylinositol GPI anchor biosynthsis, sphingolipid metabolism), high expression ACOXL was significantly enriched in metabolic pathways (linoleic acid metabolism, alpha linolenic acid metabolism) (Fig. 4H, I).

### Relationship between diagnostic biomarkers and immune cells

**Fig. 5:**
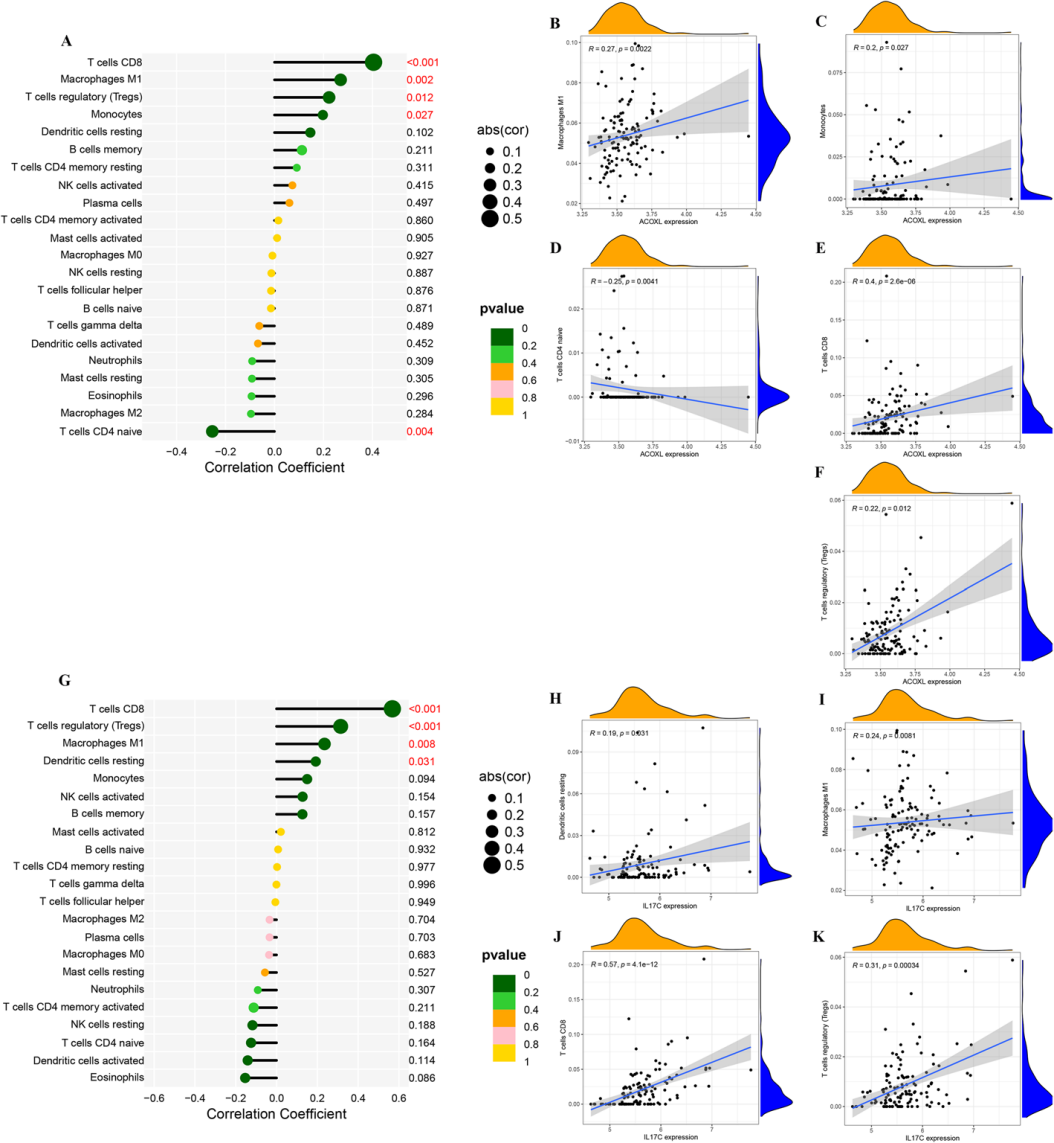
Relationship between diagnostic biomarkers and immune cells. (**A**) Correlation between 22 kinds of immune cells and ACOXL. The size of the colored squares indicates the connection’s strength. (**B**–**F**) Correlation between macrophages M1, monocytes, T cells CD8, T cells regulatory, T cells CD4 naive and ACOXL. (**G**) Correlation between 22 kinds of immune cells and IL17C. The size of the colored squares indicates the connection’s strength. (**H**–**K**) Correlation between dendritic cells resting, macrophages M1, T cells CD8, T cells regulatory (Tregs) and IL17C

Next, we investigate the role of diagnostic biomarkers in immune infiltrating of AS (Fig. 5A, G). The results showed the expression of ACOXL was positively correlated with macrophages M1 (r = 0.27, *P* = 0.002), monocytes (r = 0.20, *P* = 0.027), T cells CD8 (r = 0.40, *P* < 0.001), T cells regulatory (Tregs) (r = 0.22, *P* = 0.012), and negatively correlated with T cells CD4 naive (r = -0.25, *P* = 0.004) (Fig. 5B–F). The expression of IL17C was positively correlated with dendritic cells resting (r = 0.19, *P* = 0.031), macrophages M1 (r = 0.24, *P* = 0.008), T cells CD8 (r = 0.57, *P* < 0.001), and T cells regulatory (Tregs) (r = 0.31, *P* < 0.001) (Fig. 5H–K).

### Construction of AS diagnosis nomogram

**Fig. 6:**
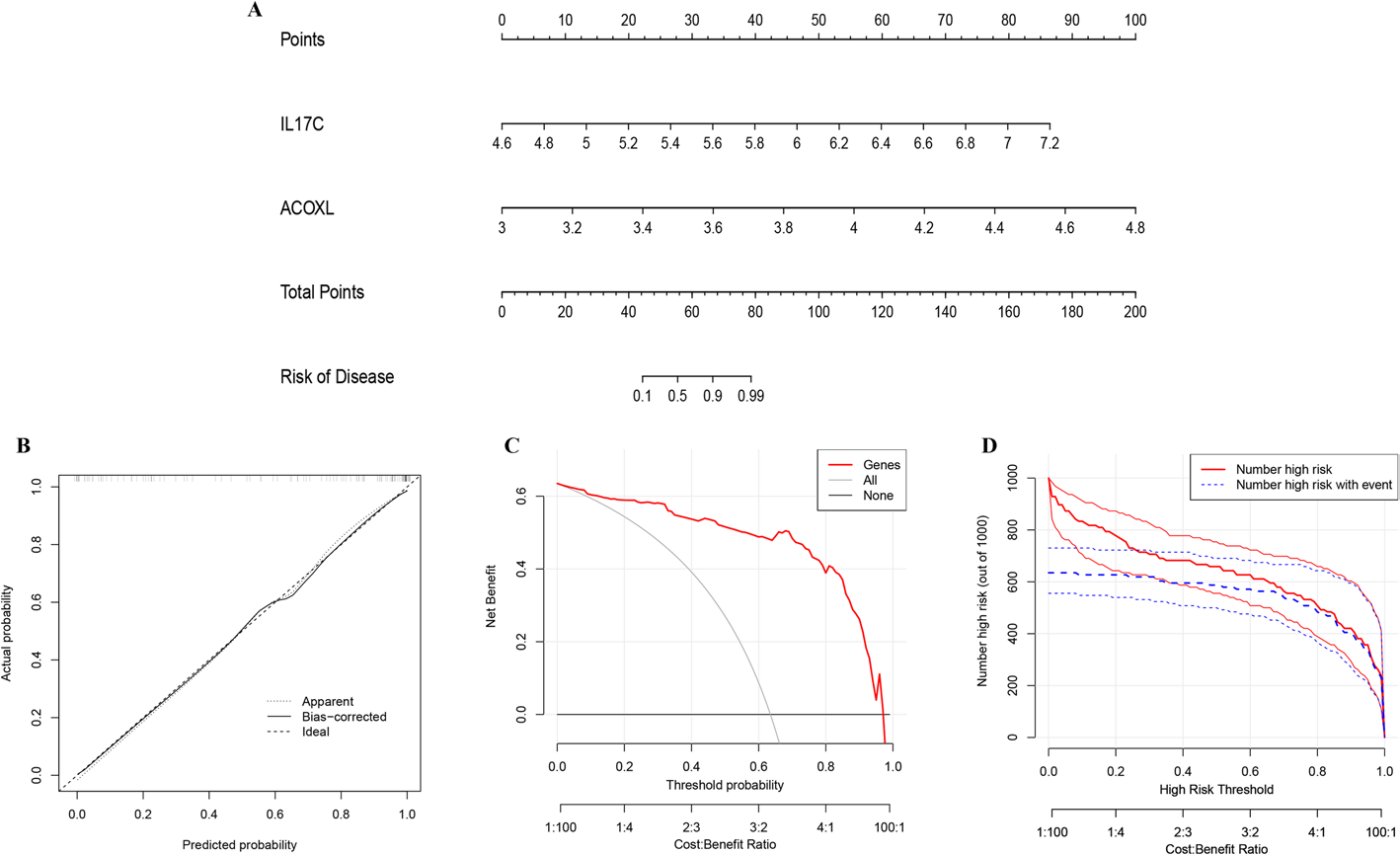
Construction of AS diagnosis nomogram. (**A**) The nomogram for predicting the risk of AS by two feature genes. (**B**) The Calibration curves of the AS prediction nomogram. (**C**) The decision curve analysis for the nomogram. (**D**) The clinical impact analysis for the nomogram

Next, we developed a diagnosis model of AS. The model that incorporated the above independent predictors was developed and presented as the nomogram (Fig. 6A). The calibration curve of the AS diagnosis nomogram for the prediction of AS risk demonstrated good agreement in this cohort (Fig. 6B). The decision curve analysis for the nomogram was presented in Fig. 6C, D. The decision curve showed that if the threshold probability of a patient and a doctor is > 1 and < 96%, respectively, using this nomogram to predict AS risk adds more benefit than the scheme.

### IL17C and ACOXL were a robust diagnostic biomarkers for AS in GSE20129, GSE43292, and GSE100927 datasets

**Fig. 7:**
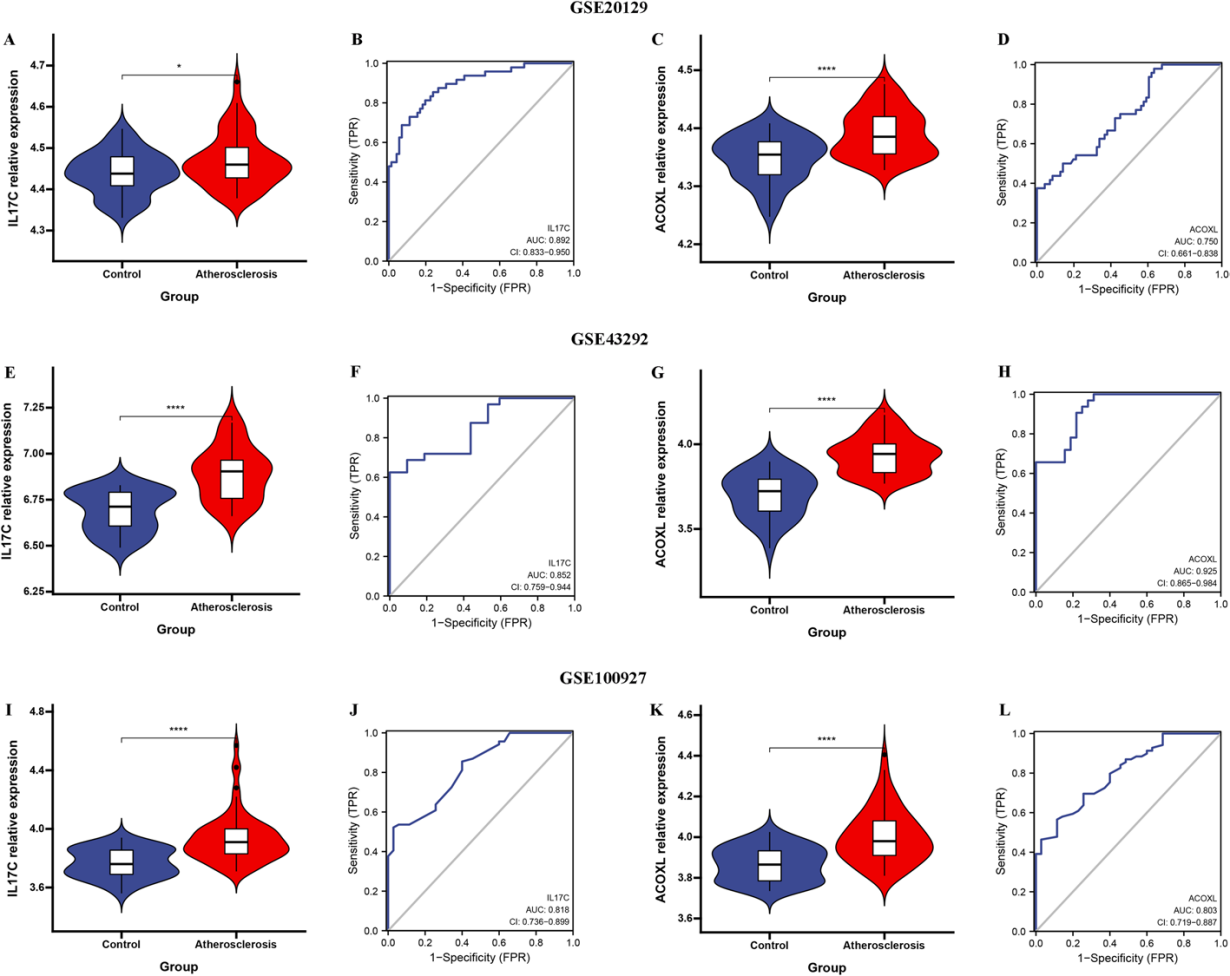
External validation of IL17C and ACOXL. The illustration shows the expression distribution of IL17C (**A**) and ACOXL (**C**) between atherosclerosis (red) and control group (blue) in GSE20129 dataset. ROC curves were constructed to assess the diagnostic accuracy of the IL17C (**B**) and ACOXL (**D**) in GSE20129 dataset. The illustration shows the expression distribution of IL17C (**E**) and ACOXL (**G**) between atherosclerosis (red) and control group (blue) in GSE43292 dataset. ROC curves were constructed to assess the diagnostic accuracy of the IL17C (**F**) and ACOXL (**H**) in GSE43292 dataset. The illustration shows the expression distribution of IL17C (**I**) and ACOXL (**K**) between atherosclerosis (red) and control group (blue) in GSE100927 dataset. ROC curves were constructed to assess the diagnostic accuracy of the IL17C (**J**) and ACOXL (**L**) in GSE100927 dataset

Consistent with this finding, increased mRNA expression of IL17C and ACOXL were observed in atherosclerosis compared with that in normal in GSE20129 datasets (Fig. 7A, C). The IL17C achieved a AUC of 0.892 (95% CI 0.833–0.950) (Fig. 7B) and the ACOXL achieved a AUC of 0.750 (95% CI 0.661–0.838) (Fig. 7D), which indicated good discrimination of the diagnostic biomarkers. Moreover, we compared the expression of IL17C and ACOXL in GSE43292 dataset, of which the expression level of IL17C and ACOXL were significantly higher in AS than in the normal tissues (Fig. 7E, G). ROC curves with AUC values was 0.852 (95% CI 0.759–0.944) for IL17C, 0.925 (95% CI 0.865–0.984) for ACOXL (Fig. 7F, H). In addition, we compared the expression of IL17C and ACOXL in GSE100927 dataset, of which the expression level of IL17C and ACOXL were significantly higher in AS than in the normal tissues (Fig. 7I, K). ROC curves with AUC values was 0.818 (95% CI 0.736–0.899) for IL17C, 0.803 (95% CI 0.719–0.887) for ACOXL (Fig. 7J, L). The above results suggest that IL17C and ACOXL were a robust diagnostic biomarkers for AS.

## Discussion

In this study, we identified two molecular subtypes based on the expression profiles, with C2 cluster showing a worse ischemic events. In addition, mRNA transcriptome differential expression genes between distinct cluster were closely related to biological processes such as immune-related pathways, epithelial cell proliferation pathways, and mitochondrial dysfunction pathways. In addition, C2 cluster had a higher T cells CD8, T cells regulatory (Tregs), M0 macrophages, and M1 macrophages, and lower M2 macrophages.

The immune system is one of the major regulatory systems in the development and progression of atherosclerosis [23]. In the early stages of atherosclerosis, low-density lipoprotein (LDL) is retained in the intima and is modified by oxidases, lipolytic enzymes, protein hydrolases and reactive oxygen species to form a variety of risk-related molecular patterns, thereby acquiring immunogenicity [24]. Immunogenic LDL activates vascular endothelial cells, which chemotacticize various immune cells into the vessel wall, mainly monocytes and T cells [25]. Histological analysis of human atherosclerotic plaques showed that M1 macrophages were mainly distributed in the lipid core, whereas M2 macrophages were mainly distributed in the plaque region away from the lipid core [26]. In vitro mouse experiments showed that M1 macrophages promote plaque inflammation, while M2 macrophages promote plaque inflammation regression [27]. We found C2 cluster had a higher M0 macrophages, and M1 macrophages, and loewer M2 macrophages, suggesting C2 cluster had a higher inflammatory environment, which leads to the progression of the disease.

CD4 + T cells receive antigens presented by antigen-presenting cells and differentiate into different Th cells (Th1, Th2, Th9, Th17, Th22, Tfh) and Treg cells through immune responses, whose role in atherosclerosis is multifaceted. Secretion of IFN-γ by Th1 cells affects macrophage polarization by inhibiting VSMC proliferation, thereby inhibiting plaque stability [28]. In addition to IFN-γ, Th1 cells secrete IL-2, IL-3, tumor necrosis factor, and lymphotoxin, all of which activate macrophages, T cells, and other cells within the plaque, thereby accelerating the inflammatory response [29]. At the same time, CD8+ T cells act on VSMC and release some inflammatory factors that make the atherosclerotic plaque unstable as well as aggravate the inflammatory response [30].

Wang et al. showed that CD68 (AUC = 0.80), PAM (AUC = 0.79), and IGFBP6 (AUC = 0.81) could be used as diagnostic markers to identify unstable plaques effectively by using LASSO and RF [31]. Xu et al. showed that C1QA (AUC = 0.83) and ITGB2 (AUC = 0.83) could be used as diagnostic markers to identify unstable plaques effectively by using LASSO [32]. In this study, multiple machine learning methods (LASSO, RF, SVM-RFE) identified IL17C (AUC = 0.92) and ACOXL (AUC = 0.90) as novel diagnostic biomarkers for atherosclerosis, and verified in other datasets.

Interleukin-17C (IL-17C) is one of the important members of the IL-17 cytokine family, which can be secreted by many types of cells or produced by the stimulation of pathogenic factors. IL-17C is mainly expressed in the mucosal surface of the gastrointestinal and respiratory tracts as well as the skin barrier. In the gastrointestinal tract, IL-17C is secreted by enteroendocrine cells and cupped cells. In the skin, it is mainly expressed by keratinocytes, monocytes and endothelial cells [33, 34]. In this present study, the expression level of IL17C was significantly higher in AS than in the normal tissues, which was consistent with previous findings. IL-17C exerts a proatherogenic effect by recruiting Th17 cells to atherosclerotic plaques [35].

Acyl coenzyme A oxidase like gene is a member of the acyl coenzyme A oxidase family. Paul et al. found that in mammals ACOXL is actively expressed at the transcriptome level, and that ACOXL is specifically expressed in the lung. ACOXL has a dehydrogenase activity of acyl coenzyme A and also catalyzes an important step in the β-oxidation pathway involving the oxidation of long-chain fatty acids [36]. Gillian et al. identified ACOXL as a biomarker for the diagnosis of prostate cancer through transcriptomics and antibody analysis of the human prostate-specific proteome [37]. In this study, we found ACOXL can be used a diagnostic biomarkers for AS, and metabolic pathways play an important role in AS disease progression. Metabolic intermediates or oxidation products produced during metabolism, such as oxidized LDL, ceramide, TMAO, and cholesterol crystals, can also be recognized by macrophages and cause activation of inflammatory pathways in the body, thus further aggravating the inflammatory response of the vasculature [38].

There are some limitations of our study. Although our analysis was based on a large sample, these cases were obtained retrospectively, and selection bias in the dataset may also affect the accuracy of the results. Large-scale prospective studies and in vivo, in vitro mechanistic studies are still needed to further confirm our results. In addition, some important clinical variables such as age, gender, and therapy information are missing in most of the datasets, we also need to combine more clinical characteristics to improve the prediction accuracy.

## Conclusions

In conclusion, we identified IL17C and ACOXL were diagnostic genes of atherosclerosis and associated with higher incidence of ischemic events. These findings may provide a new strong scientific basis for the diagnosis and treatment of atherosclerotic.

## Supplementary Information

**Additional file 1: Fig. S1**. Flowchart of dataset selecting.

**Additional file 2: Fig. S2**. Nonnegative matrix factorization (NMF) clustering was conducted and two subgroups were identified the optimal value for consensus clustering.

**Additional file 3: Table S1**. List of 223 prognostic related DEGs used in this study.

**Additional file 4: Table S2**. Identification of hub genes by using LASSO.

**Additional file 5: Table S3**. Identification of hub genes by using SVM-RFE.

**Additional file 6: Table S4**. Identification of hub genes by using RF.

## Data Availability

All data used in the study can be downloaded from the GEO database (https://www.ncbi.nlm.nih.gov/geo/query/acc.cgi?acc=GSE21545; https://www.ncbi.nlm.nih.gov/geo/query/acc.cgi?acc=GSE20129;https://www.ncbi.nlm.nih.gov/geo/query/acc.cgi?acc=GSE43292;https://www.ncbi.nlm.nih.gov/geo/query/acc.cgi?acc=GSE100927; accessed 26 May 2022).
