# Supplementary figures and images for: Nonnegative matrix factorization analysis and multiple machine learning methods identified IL17C and ACOXL as novel diagnostic biomarkers for atherosclerosis
Source: BMC Bioinformatics. 2023 May 12;24:196. doi: 10.1186/s12859-023-05244-w (PMC10176911; doi:10.1186/s12859-023-05244-w)

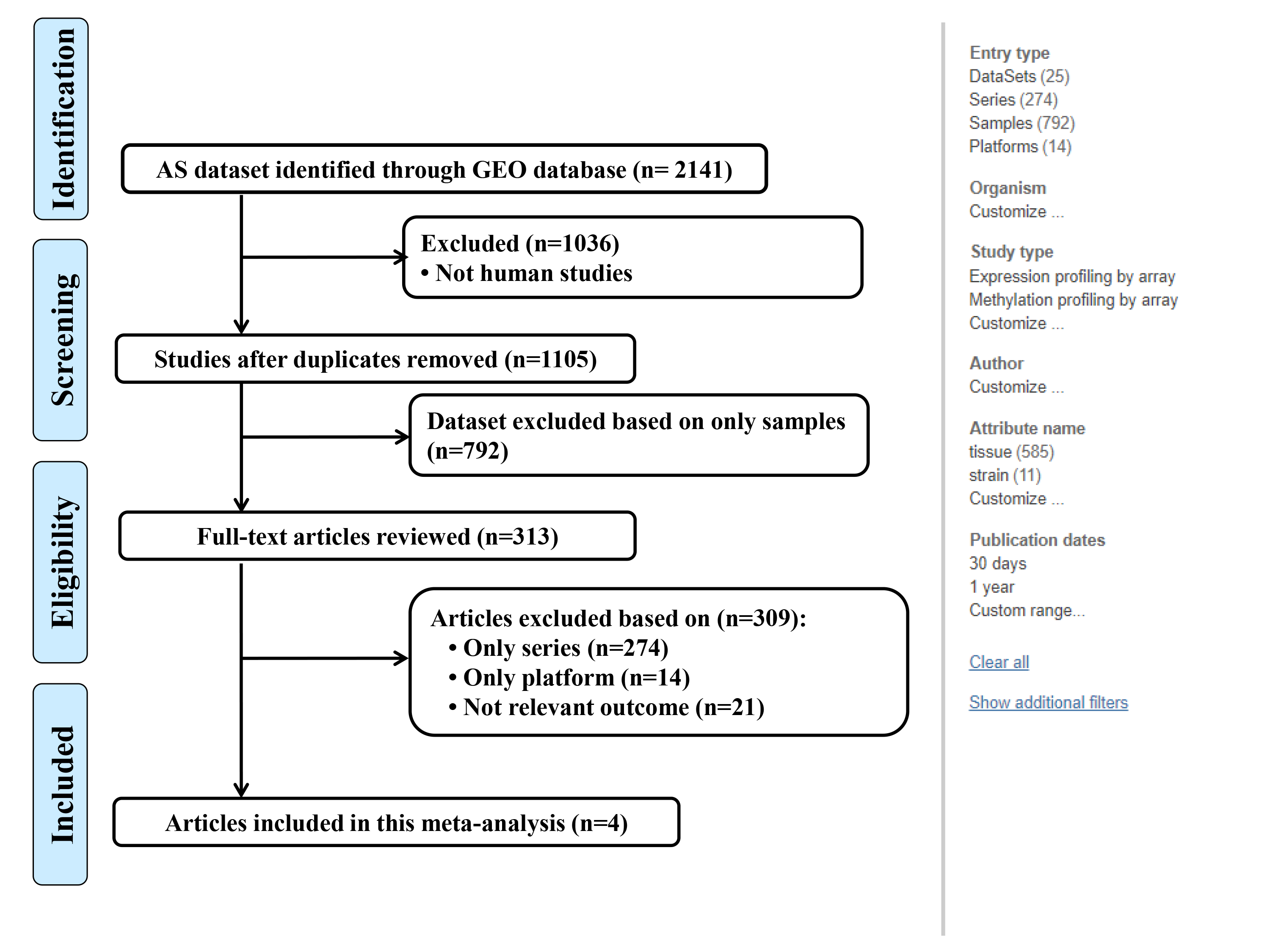

Supplement: Supplementary file 1 — Additional file 1: Fig. S1. Flowchart of dataset selecting. [file 12859_2023_5244_MOESM1_ESM.tif]

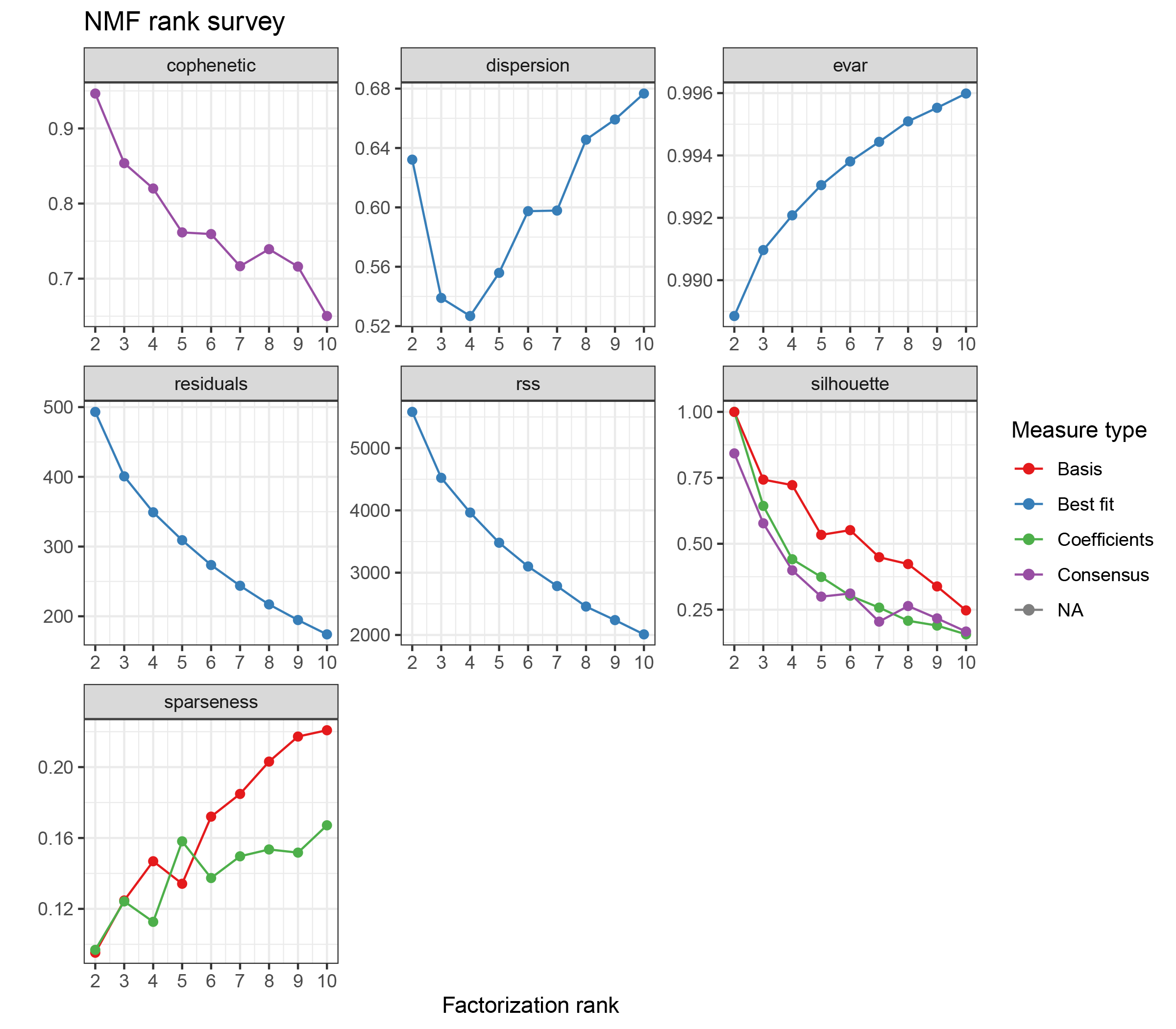

Supplement: Supplementary file 2 — Additional file 2: Fig. S2. Nonnegative matrix factorization (NMF) clustering was conducted and two subgroups were identified the optimal value for consensus clustering. [file 12859_2023_5244_MOESM2_ESM.tif]
